# General Practitioners’ Attitudes Toward a Web-Based Mental Health Service for Adolescents: Implications for Service Design and Delivery

**DOI:** 10.2196/humanfactors.8913

**Published:** 2018-03-23

**Authors:** Mirjana Subotic-Kerry, Catherine King, Kathleen O'Moore, Melinda Achilles, Bridianne O'Dea

**Affiliations:** ^1^ Black Dog Institute Randwick, Sydney Australia; ^2^ Faculty of Medicine University of New South Wales Sydney Australia

**Keywords:** anxiety, depression, adolescent, general practitioners, internet

## Abstract

**Background:**

Anxiety disorders and depression are prevalent among youth. General practitioners (GPs) are often the first point of professional contact for treating health problems in young people. A Web-based mental health service delivered in partnership with schools may facilitate increased access to psychological care among adolescents. However, for such a model to be implemented successfully, GPs’ views need to be measured.

**Objective:**

This study aimed to examine the needs and attitudes of GPs toward a Web-based mental health service for adolescents, and to identify the factors that may affect the provision of this type of service and likelihood of integration. Findings will inform the content and overall service design.

**Methods:**

GPs were interviewed individually about the proposed Web-based service. Qualitative analysis of transcripts was performed using thematic coding. A short follow-up questionnaire was delivered to assess background characteristics, level of acceptability, and likelihood of integration of the Web-based mental health service.

**Results:**

A total of 13 GPs participated in the interview and 11 completed a follow-up online questionnaire. Findings suggest strong support for the proposed Web-based mental health service. A wide range of factors were found to influence the likelihood of GPs integrating a Web-based service into their clinical practice. Coordinated collaboration with parents, students, school counselors, and other mental health care professionals were considered important by nearly all GPs. Confidence in Web-based care, noncompliance of adolescents and GPs, accessibility, privacy, and confidentiality were identified as potential barriers to adopting the proposed Web-based service.

**Conclusions:**

GPs were open to a proposed Web-based service for the monitoring and management of anxiety and depression in adolescents, provided that a collaborative approach to care is used, the feedback regarding the client is clear, and privacy and security provisions are assured.

## Introduction

### Background

Anxiety and depression are prevalent among adolescents. These disorders are associated with significant disability, including educational failure, poor relationships, and impaired daily functioning [1-3]. If left untreated, adolescents with these issues are more likely to experience chronic mental health problems in adulthood, alongside reduced workforce productivity, lower income, and poorer quality of life [4,5]. Seeking professional help is crucial for early detection, prevention, and treatment [6]. In Australia, general practitioners (GPs) provide the most accessible primary health care for young people [7,8], and are the main clinical services used by adolescents with a mental illness and emotional or behavioral problem [9]. GPs act as a first point of contact in the assessment and management of mental health issues and are well placed to identify mental health issues when youth present for other matters. GPs are a gateway to other services within the health system and facilitate access to rebated psychological therapy and psychiatric assessments. However, GPs face several barriers when treating youth mental health issues. These include inadequate training in the identification and management of adolescent mental health, a lack of confidence in recognizing youth mental health problems, and their consultation skills [10,11]. There is also a need for longer sessions, which are poorly remunerated [10,11]. Additional barriers relating to time constraints and poor linkages with other relevant services have also been reported, particularly among those in rural areas [12,13]. As such, many GPs feel ill-equipped and under-resourced when providing adolescent mental health care.

Web-based interventions, also known as e-mental health, have emerged as a safe, therapeutically effective, and acceptable referral option for common mental health concerns [14]. The advantages of using internet technology in the treatment of youth mental health have been well documented [1,15,16]. Web-based interventions are cost-effective, supplement standard therapy [17-20], and allow for greater dissemination of psychological treatments as access to the internet bypasses geographical distance, costs, and stigma [1,15,16]. Web-based interventions also improve the flexibility of psychological treatment, as they are available at a time suitable to the person and provide a sense of anonymity [21]. Two recent meta-analyses indicate that Web-based interventions are effective in reducing young people’s symptoms of anxiety and depression, and suggest that Web-based prevention and treatments are a viable alternative to face-to-face care [22,23]. In Australia, this has led to a national training initiative aimed at increasing GPs’ referrals to e-mental health. However, many GPs are still concerned about the use of eHealth. GPs cite complex systems, access issues, and privacy concerns as barriers to implementation in their practice [24,25]. Others have highlighted the lack of evidence and quality control for interventions [26], and the lack of time, technical skills, and financial compensations to implement eHealth properly [27]. In addition, youth living in rural and remote Australia may have limited access to the internet [28,29]. To address these barriers, the Black Dog Institute has designed and developed a Web-based mental health service, *Smooth Sailing*, to identify and treat anxiety and depression in secondary school students.

Delivered in the classroom, the Smooth Sailing service is based on the principles of stepped care [30], where the intensity of interventions is matched to individuals’ symptom severity. This service utilizes the internet to register students to a service where their mental health is screened. Using clinically validated algorithms, youth are automatically allocated to one of the four sequential treatment steps based on their symptom severity. Treatment intensity is matched to the step, and ranges from self-directed online psychoeducation and computerized cognitive behavioral therapy to individual face-to-face psychotherapy. The service has an e-monitoring component, in which students are sent mobile phone or email messages to assess their recent mood. In the school context, students who are at risk for suicidality or have severe levels of symptoms are automatically alerted to the school counselor who initiates consultation within 24 to 48 hours. The school counselor is then responsible for assessing risk, triaging, and referring to external support such as a GP, who can facilitate access to rebated psychological services and medication. GPs would then have access to the monitoring and alerting features. However, GPs’ attitudes toward this type of service model are unknown.

Acceptability research in health services aims to understand the extent to which people delivering or receiving an intervention consider it to be appropriate and suited to their needs, based on either anticipated or experimentation responses [31]. A study of the acceptability of the Smooth Sailing service among school counselors [32] found that personal beliefs, knowledge of e-mental health, internet accessibility, privacy, and confidentiality issues influenced their likelihood of use. Previous research on the adoption of eHealth among GPs has mainly been conducted in relation to adult health services [27]. In general, GPs and adult patients have expressed a positive attitude toward eHealth in adult health services, although GPs have also identified barriers such as time constraints, lack of skills, and lack of financial incentives to implement new ways of working [27]. In contrast, the adoption of new technologies in health care by youth mental health clinicians was less positive. Resistance to technology was based on a preference for face-to-face engagement with young consumers and a belief that the integration of new technology would create extra work in an already under-resourced environment [33]. Additional barriers identified by youth workers were related to skills, training, and concerns around confidentiality and the legal implications of online technology [33]. It is unknown whether GPs working with youth share these same concerns. Understanding the needs of GPs and their attitudes toward a Web-based, stepped care, youth mental health service is vital for designing and delivering high-quality mental health services.

### Study Aims

This study aimed to examine GPs’ attitudes toward Smooth Sailing—a Web-based, stepped care, mental health service for reducing depression and anxiety among youth. This study will help to identify the factors that may facilitate greater uptake and effectiveness of the proposed service model among GPs, and will help researchers and developers to better understand how e-mental health can be adapted for general practice.

## Methods

### Study Design

This is a multi-methods study consisting of interviews and an online survey. Ethics approval was obtained from the University of New South Wales (HC154456).

### Participants, Procedure, and Recruitment

Participants were aged over 18 years, fluent in English, and currently working as a GP in Australia. Recruitment took place between May and June 2016 via the Black Dog Institute website, social media channels, word of mouth, and an electronic direct mail-out to professional networks. The study advertisements invited GPs who were interested in the mental health of young people, the acceptability of providing mental health care to adolescents via the internet, and those wishing to inform the development of an online service. Interested participants contacted the research team and were subsequently provided with a consent form via email. Interview details were arranged via email. All interviews were conducted over the phone and were audiotaped. Interviews were conducted by one of the researchers (KOM) and were approximately 25 minutes in duration. Before the interview, a detailed overview of Smooth Sailing was emailed to participants. The interview also began with a verbal description of the service model and an opportunity to discuss and clarify any aspects of the model. The online survey link was sent to all GPs at the completion of their telephone interview. Participants were reimbursed with an Aus $20 gift voucher. Recruitment ceased when saturation was reached.

### Interview Schedule

The semistructured interview consisted of the following 4 questions:

1. What do you think of the proposed online stepped care model?
2. What do you think about the role of the GP in this model?
3. Do you have any concerns with this model and suggestions on how we could manage these?
4. What sort of things would you need to integrate this model into your general practice?

### Survey

The online survey consisted of 13 questions, with 6 questions assessing participant demographics and background factors including age, gender, state (New South Wales, Australian Capital Territory, Queensland, Victoria, South Australia, Northern Territory, Western Australia, or Tasmania), years working as a GP, mental health training (yes or no), and frequency of seeing adolescent patients (daily, weekly, fortnightly, monthly, or every 3-6 months). Participants were asked to rate the acceptability of the service (eg, *how acceptable is this online clinic to you as a GP?*) on a 5-point Likert scale ranging from unacceptable (1) to acceptable (5). In addition, participants were asked about the likelihood of integrating a Web-based service into practice (eg, *how likely are you to refer patients into the service?* and *how likely are you to integrate this service into your regular clinical practice?*) on a 5-point Likert scale ranging from not at all likely (1) to highly likely (5). Furthermore, 2 questions regarding frequency and type of feedback were asked (*If you did refer a young person, how often would you like to receive feedback about your patient’s progress?* and *What feedback would you want to receive from the online clinic?* [answered daily, weekly, fortnightly, monthly, or other]). GPs were asked what types of feedback notifications they would like to receive (registration, improvement, deterioration, suicidality, module completions, nonadherence, or other) and duration of follow-up (*once your patient has recovered, how long would you like your patient to be followed up by the service?* [answered 1-3 months, 6 months, or 12 months]) with the option of other (free response) to include alternate durations. Finally, GPs were asked if they had any other comments or advice (free response).

### Data Analysis

The online survey was delivered using the Key Survey platform version 8.13, an online survey tool developed by WorldAPP. Data were then exported to IBM SPSS version 22, and basic descriptives were calculated and reported. Audio recordings of the interviews were transcribed and analyzed using the six phases of Braun and Clarke’s [34] thematic analysis guidelines. First, two researchers (MSK and MA) read through each transcript several times to gain an overview of the interview data before code generation. Second, all data extracts were initially coded, and the search for underlying themes began by combining the emerging codes. Third, all data extracts were classified in relation to the identified themes. This process was repeated for each transcript, and any clear patterns within the data were identified. The themes were then reviewed, and any data extracts that did not fit into the initial themes were once again investigated for identification of different themes. There was discussion on higher-order codes and on points of agreement or disagreement, leading to consensual validation. Final themes were agreed upon through collaborative analysis. Final coder agreement was 80%.

## Results

### Participants

A total of 13 GPs completed the telephone interview. Of these, 11 completed the online survey and 9 out of the 11 (82%) were female. Participants had a mean age of 51.0 years (standard deviation [SD] 12.16; range: 35-79). The mean number of years that participants had been working was 21 (SD 13.76; range: 4-50). All participants had undertaken additional mental health training. Nearly half of the participants (5/11, 46%) reported having weekly contact with adolescent patients, with less than one-third (3/11, 28%) reporting daily contact.

### Level of Acceptability of the Proposed Service

All participants reported that the proposed service was entirely (9/11) or slightly (2/11) acceptable. Thematic analyses found that four key themes contributed to GPs’ acceptability of the proposed service (Table 1).

Tables 2-4 outline GPs’ preferences about the type and frequency of patient feedback and duration of follow-up within the proposed Web-based service.

As outlined in Table 5, eight themes emerged as potential barriers to the service. These themes were then classified into one of the four main issues: (1) barriers relating to the service model, (2) characteristics of youth patients, (3) environmental factors, and (4) GP characteristics.

**Table 1:** Themes contributing to the acceptability of the proposed service model (N=13).

Theme	Definition	n (%)	Example
Early intervention and prevention	The belief that the service provides an opportunity to detect youth with subthreshold symptoms who may remain undetected or receive little attention by traditional services, thus providing early intervention	7 (54)	*I think it’s useful to be universally screening adolescents and it’s useful to be capturing this subsyndromal group early who generally would not receive any attention. I think the potential to intervene at that point and prevent further escalation is really important.* [GP1]
Adolescent preferences	The belief that the service was an appropriate platform for this age group because of adolescent preferences for technology and spending time online	8 (62)	*...adolescents these days are completely plugged into technology and I think it’s giving them access to assessment and treatment via a platform that they’re comfortable with.* [GP5]
School context	The belief that the service would lead to greater access of care because it is free, online, delivered in the school environment, and complements traditional modes of therapy (eg, face-to-face)	8 (62)	*My experiences there are that school counselors are very busy...So, it’s great for them to be able to actually say well look here’s something that you can do in the meantime.* [GP4]
Anonymity	The belief that this type of service would provide anonymity, reducing stigma and ensuring privacy	5 (39)	*I think that they prefer it has a level of anonymity, which is great, and it doesn’t necessarily involve parents or care providers having to know what’s going on in your life, so I think it’s a great idea for young people.* [GP5]

**Table 2:** Type of patient feedback desired by general practitioners (N=11).

Type of feedback notifications	n (%)
Patient registration to online service	9 (82)
Clinical improvement	10 (91)
Clinical deterioration	11 (100)
Suicidality	11 (100)
Number and type of modules completed	6 (55)
Ceasing use	10 (91)
Other: information about referral pathways, who the general practitioner has permission to speak to (eg, caregivers and health professionals); child protection or domestic violence issues; and details of specific areas covered in online modules	3 (27)

**Table 3:** Frequency of patient feedback desired by general practitioners (N=11).

Frequency of feedback notifications	n (%)
Daily	2 (18)
Weekly	0
Fortnightly	3 (27)
Monthly	3 (27)
Other: frequency should decrease over time; should be dependent on client progress or step allocation or symptom severity	3 (27)

**Table 4:** Duration of follow-up desired by general practitioners (N=11).

Duration of follow-up	n (%)
1 to 3 months	2 (18)
6 months	4 (36)
12 months	3 (27)
Other: depends on duration of involvement and time taken to recover; should be determined by the clinical presentation and patient	2 (18)

**Table 5:** Potential barriers to the proposed service model (N=13). GPs: general practitioners. CBT: cognitive behavioral therapy.

Issues and theme		Definition	n (%)	Example
**Service model**				
	Confidence and knowledge of eHealth	The degree to which GPs thought that there was a shortage of evidence-based online care and the personal preference for face-to-face treatment	11 (85)	*I think one of the issues with online services for young people is a lack of evidence...* [GP13]
	Privacy and confidentiality	The degree to which GPs were concerned with data storage, access, and privacy between GPs, school counselors, and caregivers (eg, data being mishandled or accessed without authorization)	6 (46)	*I think the important thing in that context is to make sure that there’s an area where students can go and access the program, maybe somewhere that’s quiet or separate and which respects their confidentiality as I think certainly confidentiality and privacy is a huge issue.* [GP7]
	Effectiveness and accuracy of the proposed model	The degree to which GPs were concerned about youth not receiving appropriate care	5 (38)	*I guess the only concern one could ever have is that someone who’s severely unwell doesn’t end up with their matching care.* [GP11]
**Characteristics of youth patients**				
	Noncompliance from adolescents	A concern that there may be a risk that some young people do not engage with the program and motivation to complete the online modules may be low	8 (62)	*I have been aware of online CBT courses that are available, and my uptake is really bad when I suggested it to patients...adolescents, young adults; even when they’ve been enthusiastic, they don’t generally follow through with it.* [GP13]
**Environmental factors**				
	Access issues	Potential constraints regarding availability of these services (eg, internet and phone access, and rural location) and client characteristics (eg, the inability to access service because of learning difficulties, cultural barriers, low school attendance, or complex clinical presentations)	5 (38)	*Kids that live remote...their lack of access and connectivity, no Wi-Fi and a lot of kids have got phones but they’re all the old phones...not smartphones.* [GP8]
				*...the kids that you want to target...They’re the kids who actually aren’t going to school or who’re very haphazard, who are not staying at home anymore for whatever reason.* [GP8]
	Lack of services	Limited availability of appropriate services in rural and remote areas (eg, limited options for referral, lack of qualified professionals, and adolescent specific services)	4 (31)	*I work in a rural location, and in reality, I have access to very, very few services and the services that are available are usually targeted to adults...*[GP6]
**GP characteristics**				
	Differences amongst GPs	Perceived differences in GPs’ mental health knowledge and experience with adolescents	5 (38)	*With increased numbers of bulk-billing clinics, you’re not going to get GPs with complete GP training or mental health training...*[GP11]
	Noncompliance by GPs	Risk that GPs will not use service or have no incentive to use the service (ie, without rebate or ability to bulk bill for time spent outside direct consultation)	3 (23)	*The remuneration you get isn’t there...because we can only charge Medicare when we’ve got the patient with us...so, you know when I ring the school afterward or when reading reports, writing reports, stuff like that—there isn’t a financial incentive.* [GP8]

### Likelihood of Referral and Integration Into Clinical Practice

All GPs reported that they were highly (7/11) or somewhat (4/11) likely to refer youth patients to the proposed Web-based service. Thematic analyses found that two themes influenced likelihood of referral (Table 6).

All GPs reported they would be highly (6/11) or somewhat (5/11) likely to integrate the proposed Web-based service into their clinical practice. Thematic analyses revealed that six key needs influenced the likelihood of GPs integrating the proposed service (Table 7).

**Table 6:** Themes influencing likelihood of referral of the proposed Web-based service (N=13). GPs: general practitioners.

Theme	Definition	n (%)	Example
Perceived need	Whether the GPdentified a need for the service	11 (85)	*We’re all buckling under the strain with the amount of work to do not just with adolescents but with mental health in general and anything that will help management and prevention is a fantastic tool.* [GP12]
Beliefs	Whether the GP felt the service would be helpful, promote help-seeking, or support clinical practice	4 (31)	*It sounds like the system allows regular monitoring and safeguards to the GP, then it certainly helps the GP to really manage the person.* [GP9]

**Table 7:** General practitioner (GP) needs for service integration (N=13).

Need	Definition	n (%)	Example
Collaborative approach to management of patient	A belief that the service was likely to be integrated if a coordinated approach between adolescents, caregivers, school counselors, GPs, and other health professionals is adopted	10 (77)	*I think that a shared care model is excellent with everybody being on the same page is vital.* [GP10]
Duty of care and medico-legal implications	The need for delineation about who is part of the team and who is responsible for responding to alerts. Informed consent, transparent information about client progress, and a user-friendly feedback system were also discussed. GPs also outlined the need for legal advice (eg, notifying parents, consent, and medico-legal responsibility) and mandatory reporting guides (eg, self-harm, underage sex, substance use, and domestic violence)	8 (62)	*I think GPs are going to have questions like, so what am I going to do if I get a message from you that says the patient is deteriorating or that says the patient has stopped using the program; is it my medico-legal responsibility to follow the patient up?...those kinds of questions, GPs are going to want to know the answers to.* [GP2]
Encourage relationship with GP	A belief that the program should encourage adolescents to build a relationship with a GP, for example, include information about how to find a GP, the types of issues a GP can assist with, and help adolescents with appointments	10 (77)	*...having a teenager build a relationship with a GP almost needs to be a goal within this, to work toward them having their own GP maybe separate to their parents and to understand how to use their relationship with the GP...* [GP1]
Quality of service information provided to GPs	The need for concise information provision, for example, specific details about how the service works and links to relevant information as a reference for GPs	8 (62)	*...some sort of clear and detailed summary that explains some of the skills and the strategies that people are learning will help me to understand what it is that I’m referring people to.* [GP7]
Community awareness and promotion of the service	Strategies to overcome a lack of community awareness, for example, promotion in schools, advertising in newspapers, and promotion in health centers by health professionals	11 (85)	*...I’d need like some sort of way of referring the patients, so whether it’s sort of a brochure to hand over, or whether it’s card with the email or online link or whether it’s a referral pad.* [GP10]
Training	A request for training for GPs and school counselors on how to use the service and provide feedback	6 (46)	*I think an educational video would be really good...* [GP7]

## Discussion

### Principal Findings

This study aimed to examine GPs’ attitudes toward a Web-based mental health service for treating depression and anxiety among adolescents. This study also aimed to identify barriers to integrating a Web-based service into clinical practice. This knowledge is important for determining best practice in the field of e-mental health service design and delivery for youth. In this study, most GPs reported that the proposed service was an acceptable type of care because of its focus on early intervention and prevention, the alignment with adolescents’ help-seeking preferences, the provision of anonymity to reduce stigma and ensure privacy, and its delivery in the school setting. These attitudes are consistent with other health workers, who have reported that access to care via the internet overcomes geographical, psychological, and physical barriers, is cost-effective, and a viable adjunct to standard therapy [1,15,16,35,36]. The sense of anonymity of online care somewhat overcomes issues relating to stigma and embarrassment about seeking help for mental health problems, which has previously been identified as the most prominent barrier to help-seeking in young people [37]. The stepped care component of the model was also supported. GPs felt that the proposed service could improve the detection of mental health problems among youth and identify those with subthreshold symptoms who are currently not receiving care. Future evaluations of the service will need to provide evidence to support the effectiveness of such a model in this severity group.

### Factors Influencing Likely Use

In this study, the GPs identified several issues that may influence the acceptability, both positively and negatively, of the proposed Web-based service. Participants reported that GPs may be concerned that young people using a Web-based service may not receive adequate care, or that some youth may not be able to access the service because of poor internet connectivity. In addition, participants reported that GPs may be worried about data security and privacy. Although this is a common concern of internet-delivered care [24,25,32], participants felt that this could be alleviated by clear delineation of roles and responsibilities within the service alongside robust informed consent procedures with the young person. Additional barriers to acceptability of the proposed service included noncompliance by adolescents and GPs, as well as differences in mental health training and youth experience of GPs. Interestingly, lack of financial incentives for extra consultation time was only mentioned by 3 GPs. This is in contrast to previous research that identified poor financial reimbursement as a major constraint to GPs delivering high-quality youth mental health care [10,13]. These differences in study findings may be because of unique characteristics of the Web-based service (eg, the school counselor being the primary carer, thus reducing the workload of the GP) or because of characteristics of the GPs interviewed (eg, all reported having mental health training, thus the service proposed may not be viewed as an additional burden on time). However, this is purely speculative and future studies would benefit from investigating whether financial incentives increase rates of referral among GPs.

### Importance of Patient Follow-Up and Feedback

All GPs agreed that they would like feedback on symptom deterioration and suicidality, as well as clinical improvement. This is not surprising, given the significant ethical and legal obligations associated with providing treatment to adolescents. Interestingly, perspectives on the frequency of feedback and duration of follow-up varied. Half of the GPs surveyed wanted fortnightly or monthly feedback, whereas others felt feedback should be dependent on patient progress and/or symptom severity. Over half of the sample felt the duration of follow-up should not exceed 6 to 12 months. These results highlight the importance of monitoring functionality, and a future model of the service should incorporate customizable options for frequency of patient feedback and follow-up to account for GP preferences.

### Likelihood of Referral and Integration Into Clinical Practice

All GPs reported that they were highly or somewhat likely to refer youth patients to the proposed Web-based mental health service. However, referral was found to be influenced by perceived need and beliefs, such that GPs who do not self-identify as needing assistance in providing care to youth, or those who do not believe in the effectiveness of Web-services, would be unlikely to refer to the proposed service. This is consistent with previous research in which GPs’ attitudes to treatments were largely influenced by their personal beliefs about effectiveness [26,33]. This poses a significant challenge to researchers and policy makers who are attempting to increase GPs’ use of e-mental health. A future trial of the proposed service may need to involve additional pretraining that addresses GP's knowledge and awareness of e-mental health.

Although the participants reported that they would be likely to integrate the proposed service model into their clinical practice, six key needs were outlined. GPs were more likely to integrate the proposed service if it involved close collaboration with other health care professionals, duty of care and medico-legal implications were clearly addressed, the service encouraged an ongoing relationship with a GP, the quality of information provided to GPs about the service was concise, and if community awareness and promotion of the service, as well as training on how to use the service, was offered. Future trials of the service will need to ensure that GPs have mechanisms of maintaining contact with school counselors, parents, and any additional care providers involved in the treatment of the client. Trial studies of the service may benefit from demonstrations of the online service, alongside case studies and evaluation data to increase the likelihood of GPs recommending and using the service.

### Limitations

Although this study provides support for the acceptability of a Web-based mental health service for adolescents by GPs across Australia, there were a few limitations. First, it is a small study in which only 13 participants were involved. These findings therefore present only an initial indication of GPs’ attitudes, which would be strengthened by a larger sample size. GPs in this study were not using the proposed service. Thus, the results indicate intention rather than actual behavior. In addition, all participants had undergone additional mental health training and had experience working with young people. Different results may have been found among GPs without this, and future work may be strengthened by targeting different GPs with less training and youth experience. Views may also differ among international samples, where the role of GPs in adolescent mental health care may vary.

### Conclusions and Future Work

Overall, GPs in this study were open to the proposed service, but have concerns about certain aspects of Web-based care, characteristics of youth and GPs, and accessibility of a Web-based service. These would need to be addressed before GPs will refer or integrate the service into their clinical practice. In future work, researchers and service designers need to consider GPs as end users and evaluate the effects of the proposed service model on GPs’ confidence in delivering youth mental health care and the clinical effectiveness. Key next steps would be a pilot study of the service in primary care settings, alongside a formal evaluation of the effectiveness of the service for improving quality of care and symptom reductions among youth. Given the complex nature of the intervention and its setting, the multiphase optimization strategy methodology [38], utilizing factorial designs, may be well suited to evaluating the independent effects of each of the components. This will assist the service designers and developers to optimize the service for both the practitioner and the patient.

## Abbreviations

CBT: Cognitive Behavior Therapy
GP: General practitioner
